# Effects of High Pressure Homogenization on the Activity, Stability, Kinetics and Three-Dimensional Conformation of a Glucose Oxidase Produced by *Aspergillus niger*

**DOI:** 10.1371/journal.pone.0103410

**Published:** 2014-07-25

**Authors:** Alline Artigiani Lima Tribst, Júnio Cota, Mario Tyago Murakami, Marcelo Cristianini

**Affiliations:** 1 Department of Food Technology (DTA), School of Food Engineering (FEA), University of Campinas (UNICAMP), Campinas, SP, Brazil; 2 Department of Food Science (DCA), School of Food Engineering (FEA), University of Campinas (UNICAMP), Campinas, SP, Brazil; 3 Brazilian Bioethanol Science and Technology Laboratory (CTBE/CNPEM), Campinas, SP, Brazil; 4 Brazilian Biosciences National Laboratory (LNBio/CNPEM), Campinas, SP, Brazil; Institute of Enzymology of the Hungarian Academy of Science, Hungary

## Abstract

High pressure homogenization (HPH) is a non-thermal method, which has been employed to change the activity and stability of biotechnologically relevant enzymes. This work investigated how HPH affects the structural and functional characteristics of a glucose oxidase (GO) from *Aspergillus niger*. The enzyme was homogenized at 75 and 150 MPa and the effects were evaluated with respect to the enzyme activity, stability, kinetic parameters and molecular structure. The enzyme showed a pH-dependent response to the HPH treatment, with reduction or maintenance of activity at pH 4.5–6.0 and a remarkable activity increase (30–300%) at pH 6.5 in all tested temperatures (15, 50 and 75°C). The enzyme thermal tolerance was reduced due to HPH treatment and the storage for 24 h at high temperatures (50 and 75°C) also caused a reduction of activity. Interestingly, at lower temperatures (15°C) the activity levels were slightly higher than that observed for native enzyme or at least maintained. These effects of HPH treatment on function and stability of GO were further investigated by spectroscopic methods. Both fluorescence and circular dichroism revealed conformational changes in the molecular structure of the enzyme that might be associated with the distinct functional and stability behavior of GO.

## Introduction

Glucose oxidase (GO) (β-D-glucose:oxygen 1-oxidoreductase) (EC 1.1.3.4) is a dimeric glycoprotein, consisting of two polypeptide chains covalently linked by disulfide bounds [1]. The enzyme has a FAD as cofactor, which is covered by a lid formed by a two-stranded antiparallel β-sheet that prevents the release of the cofactor from the dimer [2], [3]. The GO dimer is stabilized by six salt bridges and 15 hydrogen bonds, eight of them to the carbohydrate moiety [3]. The enzyme catalyzes the oxidation of β-D-glucose to gluconic acid using molecular oxygen as the electron acceptor, with simultaneous production of hydrogen peroxide [1], [4], [5]. The enzyme can be produced by yeasts and molds, and *Aspergillus niger* is the main microorganism used for GO production [6].

GO has been employed to remove oxygen from food, improving its color, flavor and shelf life. It can also be applied to the removal of glucose from eggs before pasteurization or drying, avoiding browning by the Maillard reaction [1]. Furthermore, it has been increasingly used in analytical methods, particularly those for quantitative determination of D-glucose in samples such as body fluids, foodstuffs, beverages and in fermentative processes [1].

GO is an unstable enzyme, being denatured at temperatures above 60°C, at pH values higher than 6.0 or lower than 4.0 and also in aqueous solution, with a half-life of 30 minutes [1]. Thus, strategies must be developed to enhance GO stabilization and, consequently, to improve its commercial applications.

HPH technology – also known as dynamic high pressure [7] and ultra-high pressure homogenization [8] – is an emerging technology developed for food preservation, showing minimal nutritional and sensory damage [9], [10]. This process was previously studied to inactivate microorganisms [8], [9], [11] and to cause changes in the structure of proteins, polysaccharides and suspended particles [7], [12], [13].

The effects of HPH on enzymes have been studied by a few authors [7], [14]–[23]. Some of these authors found changes in the conformation of the enzymes [16], [22], resulting in enzyme activation [15], [16], [18], [19], [20], [22], [23], thermal stabilization [24] or inactivation [7], [14]. However, this process did not affect α-amylase activity [17]. Therefore, the effect of high pressure homogenization is dependent on the type of enzyme, the level of pressure evaluated, the number of passes and the presence of substrate during homogenization. Previous results obtained for a commercial GO indicated that HPH improved the enzyme activity at high temperatures (75°C) and also enhanced enzyme stability after one day of storage under specific conditions [19]. These results suggest that HPH could be applied as a tool to improve the characteristics of GO, and consequently increase the application range of GO. Considering previous data, this work was performed to evaluate how the process changed the activity, stability and kinetic parameters of GO and to correlate these changes with HPH-induced alterations in the structural conformation of GO.

## Materials and Methods

### Enzyme characteristics

The glucose oxidase used in this experiment was a highly pure grade enzyme from Amresco, obtained by fermentation with *Aspergillus niger* (AMRESCO, USA, Code 0243-100 KU, batch number 1411C338). The enzyme has ∼160 kDa and 16% of carbohydrates. The purity grade of glucose oxidase was 99%.

### Enzyme activity

The activity of GO was determined using a method previously described [19]: 400 µL of enzyme solution (0.05 g of dried enzyme per liter of 0.1 M acetate buffer, pH 5.0 plus 0.02 g.L^−1^ of sodium nitrate) were added to 400 µL of glucose solution (4 g.L^−1^) prepared in the same buffer and to 1.2 mL of 0.1 M acetate buffer pH 5.0. The reaction was carried out at 50°C for 30 minutes. An aliquot of 1.5 mL of 3,5-Dinitrosalicylic acid (DNS) solution [25] was then added followed by heating at 100°C for 5 minutes to stop the reaction. A blank buffer was obtained using a similar procedure, but without the addition of the enzyme. After heating, the samples were cooled and 6.5 mL of 0.1 M acetate buffer (pH 5.0) was added. Absorbance was measured at 547 nm in a DU 800 spectrophotometer (Beckman Coulter, California, USA).

The standard curve was obtained using glucose solutions at concentrations of 0.5, 1, 2, 3, 4, 5, 6, 7, 8, 9 and 10 g.L^−1^ prepared in 0.1 M acetate buffer, pH 5.0. Glucose was measured in triplicates according to the above described DNS assay. The measured absorbance was converted into glucose concentration unit using the standard curve. The activity of GO was calculated from the difference in glucose concentration between the control and the GO samples. One enzyme unit was defined as the amount of enzyme which converts 1 µg of glucose per minute. The final GO activity was calculated per gram of dried enzyme. The enzyme unit was calculated as described in equation 1:
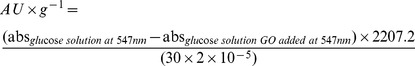
(1)


Where:

2207.2 was the slope of the glucose standard curve of absorbance vs. concentration
30 is the time (in minutes) of reaction
2*10^−5^ is the amount of enzyme (in grams) added in the reaction tube

### High pressure homogenization

A Panda Plus High-Pressure Homogenizer (GEA-Niro-Soavi, Parma, Italy) was used in the assays. This equipment has a single acting intensifier pump that amplifies the hydraulic pressure up to 200 MPa and operates at a flow rate of 9 L.h^−1^.

A volume of 2 L of the GO solution (0.05 g.L^−1^) at pH 5.0 was homogenized under pressures of 75 and 150 MPa, using an inlet temperature of 25.6°C. Samples (50 mL) were collected and cooled in an ice bath and the unprocessed sample of GO (native) was defined to be a control, as previously described [19].

### The effects of pH on the activity of native and homogenized GO

The activity of GO was evaluated at pH 4.5, 5.0, 5.5, 6.0 and 6.5, using 0.1 M acetate buffer (pH 3.5–5.5) and 0.1 M citrate-phosphate buffer (pH 6.0–6.5). The enzyme activity was measured by the DNS method, changing the pH of the buffer and using temperatures of 15, 50 and 75°C to determine the activity. Standard curves were obtained using glucose solutions at concentrations of 0.5–6 g.L^−1^ prepared in the same conditions.

The optimum pH was chosen considering the highest activity of the native (unprocessed) sample. Under this condition (optimum pH), 100% of relative activity was established. For the other samples evaluated (including HPH-treated ones), the relative activities were calculated using equation 2.
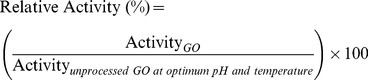
(2)


### Effects of the temperature on GO activity

The activity of GO was evaluated under different temperatures (15, 25, 35, 45, 50, 55, 65, 75 and 85°C). Enzyme activity was measured by the DNS method at pH 5.0 (optimum pH), changing the temperature of the enzyme reaction. The optimum temperature was chosen considering the highest activity of the native sample as measured in the experiment. Under this condition (optimum temperature), 100% of relative activity was established. For the other samples, the relative activities were calculated using equation 2.

### GO stability at 60°C

The stability of GO was assessed by incubating the GO solution in tubes at 60°C for 40 minutes, taking samples at 5 minute intervals and cooling them down in an ice bath. The activity was immediately measured by the method described in section 2.2. The residual activity after each residence time at 60°C was determined using equation 3 and the initial activity for each sample determined just after the lag time (time required for the samples to reach 60°C at the center of the tube = time 0).
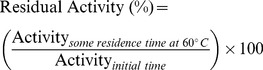
(3)


### Stability of the GO solution under refrigerated storage

Samples of native and homogenized (75 and 150 MPa) GO solutions were stored for 24 h under refrigeration (8°C) and the activity of GO then determined at 15, 50 and 75°C using the DNS method. The relative activities of the samples were calculated using equation 2 and the results compared with those obtained immediately after homogenization (0 day).

### Kinetic parameters of GO

GO kinetics was assayed using glucose as the substrate at concentrations of 0.2, 0.4, 0.8, 1.2, 1.6, 2.0, 2.4, 2.8, 3.2, 3.6 and 4.0 g/L. The reaction was carried out according to the methodology described in section 2.2, changing the concentration of the glucose solution. To obtain the Michaelis-Menten constant (K_m_) and maximum velocity (V_max_), the kinetic data were plotted as the velocity of GO activity (measured in glucose conversion.min^−1^) versus the glucose concentration. The kinetic parameters (K_m_ and v_max_) of the Michaelis-Menten model were obtained by nonlinear regression using the software CurveExpert Professional (v.1.5, http://www.curveexpert.net/, USA) with a significant probability level of 95%.

### Circular Dichroism

CD spectra were recorded using a JASCO J-715 CD spectropolarimeter (JASCO, Tokyo, Japan) with a 0.1 cm quartz cuvette at room temperature. CD measurements were scanned in the far-UV range (250–198 nm) with 8 replicates at a rate of 100 nm.min^−1^. The band width was 1.0 nm. CD data were expressed in terms of molar ellipticity (θ) in deg.cm^2^.dmol^−1^ [24]. For CD analysis, sample concentration was adjusted to 0.4 mg.mL^−1^. The values of 8 replicates were averaged and the curves were smoothed using the JASCO Spectra Manager II software according to the fast Fourier transform noise reduction routine. Thermal unfolding experiments were monitored at 220 nm and the sample was heated at a rate of 1°C/min. Data analysis was performed according to methods previously described [26], [27], [28].

### Fluorescence spectroscopy

Intrinsic emission fluorescence spectra of glucose oxidase samples were measured using a FS F-4500 spectrofluorometer (Hitachi Ltd, Japan) with a slit width of 5 nm. The GO solutions (500 mg.L^−1^) were processed at 75 and 150 MPa and a native sample was used as the control. To minimize the contribution of tyrosyl residues to the emission spectra, GO solutions were excited at 280 nm, and emission spectra were recorded from 300 to 400 nm [24].

### UV-absorption

The UV absorption spectra of native and homogenized GO solutions (500 mg of GO prepared in one liter of 0.1 M acetate buffer pH 5.0) were obtained using an UV–VIS DU 800 spectrophotometer (Beckman Coulter, Brea, CA) and a scanning range from 200 to 400 nm [24].

### Statistical analysis

Analysis of variance (ANOVA) was applied to compare the effects of the different treatments, and the Tukey test was used to determine the differences between them at a 95% confidence level. The statistical analyses were carried out using the STATISTICA 5.0 software (StatiSoft, Inc., Tulsa, Okla., USA). All the processes and the determination of glucose oxidase activity were carried out in triplicate. The results were represented as the mean ± standard deviation.

## Results and Discussion

### Effects of HPH treatment on GO activity

The enzyme activity was measured at temperatures ranging from 15 to 85°C and at pH values ranging from 4.5 to 6.5. The maximum activity (3,224,591 U.g^−1^), defined as 100%, was determined at pH 5.0 and 50°C. These results were subsequently compared with the HPH-treated enzyme to evaluate if the process promoted changes in the enzyme activity under optimum and non-optimal conditions.

**Figure 1:**
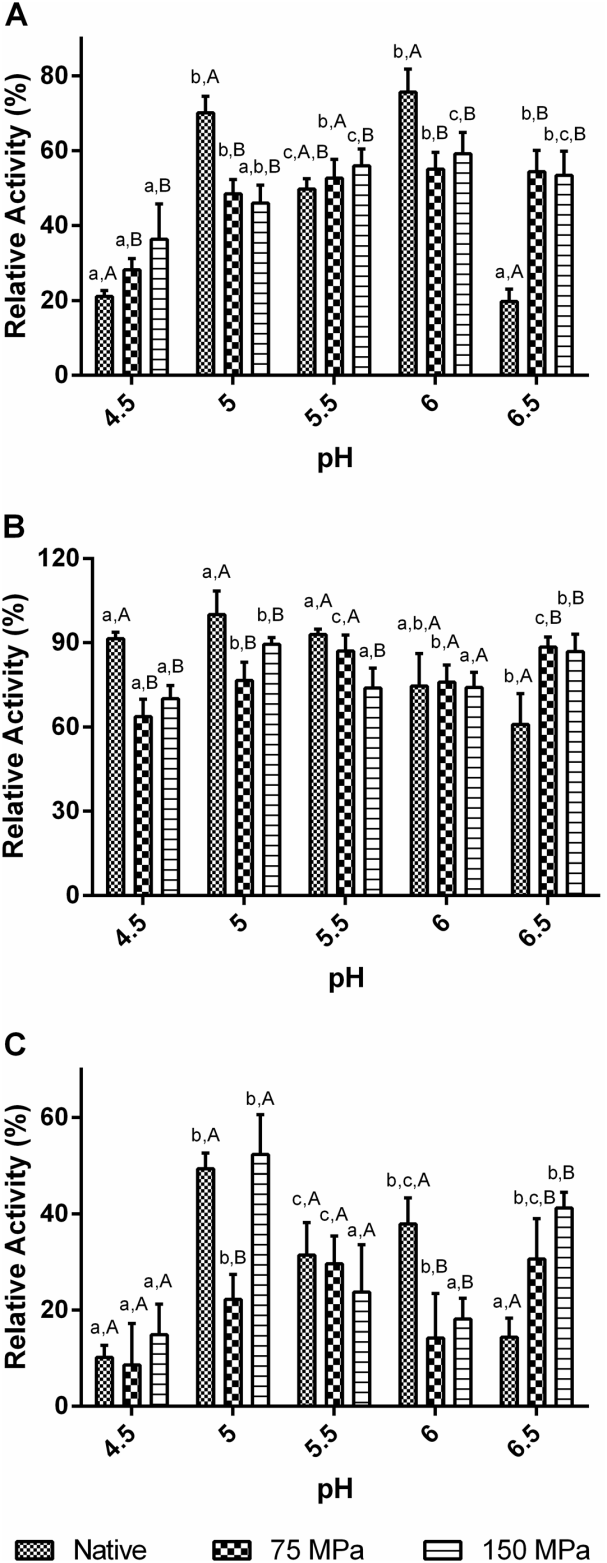
Activity of the native and HPH-treated GO samples at different pH and temperatures 15°C (A), 50°C (B) and 75°C (C).

The HPH treatment was carried out at 75 and 150 MPa, considering previous data [19], in which were observed significant improvements in activity at high temperatures and after a storage period for a commercial glucose oxidase treated at 150 MPa. Figure 1 shows the results obtained for the activities of the native and homogenized enzymes at pH values from 4.5 to 6.5 and temperatures of 15 (Figure 1A), 50 (Figure 1B) and 75°C (Figure 1C).

The results at 50°C showed that the native enzyme maintained high activity levels in the evaluated pH range (minimum activity around 60% at pH 6.5). At 15°C and 75°C, pronounced activity loss was observed in the extremes of the pH range (pH 4.5 and 6.5), indicating that both pH and temperature play an important role in the maintenance of GO structure and its ability to react with the substrate.

The analysis of enzymatic activity assays shows that there was no difference among HPH treatments using 75 and 150 MPa. Perhaps it indicates that the energy supplied to enzyme at 75 MPa was enough to induce conformational changes in protein structure, which affected the enzymatic activity of GO.

Comparing the results for native and HPH-treated enzymes, a significant loss in GO activity was observed in the majority of conditions under which the activity was measured. The main exceptions were observed at the extreme pH values within the evaluated range (4.5 and 6.5). At lower pH, the enzyme activity remained the same (75°C) or increased (15°C) after the HPH process. At pH 6.5, a considerable improvement in activity was observed for all the temperatures used to measure activity. Therefore the results demonstrated that HPH broadened the pH range of GO activity, especially at high pH values. This might be very useful in the food industry for the application of GO to reduce the glucose concentration in liquid egg before pasteurization, since the product has a neutral pH [1]. In addition the activity at pH 6.5 may help to improve the use of the enzyme as a biosensor of biological fluids, such as blood and urine [1], [5].

**Figure 2:**
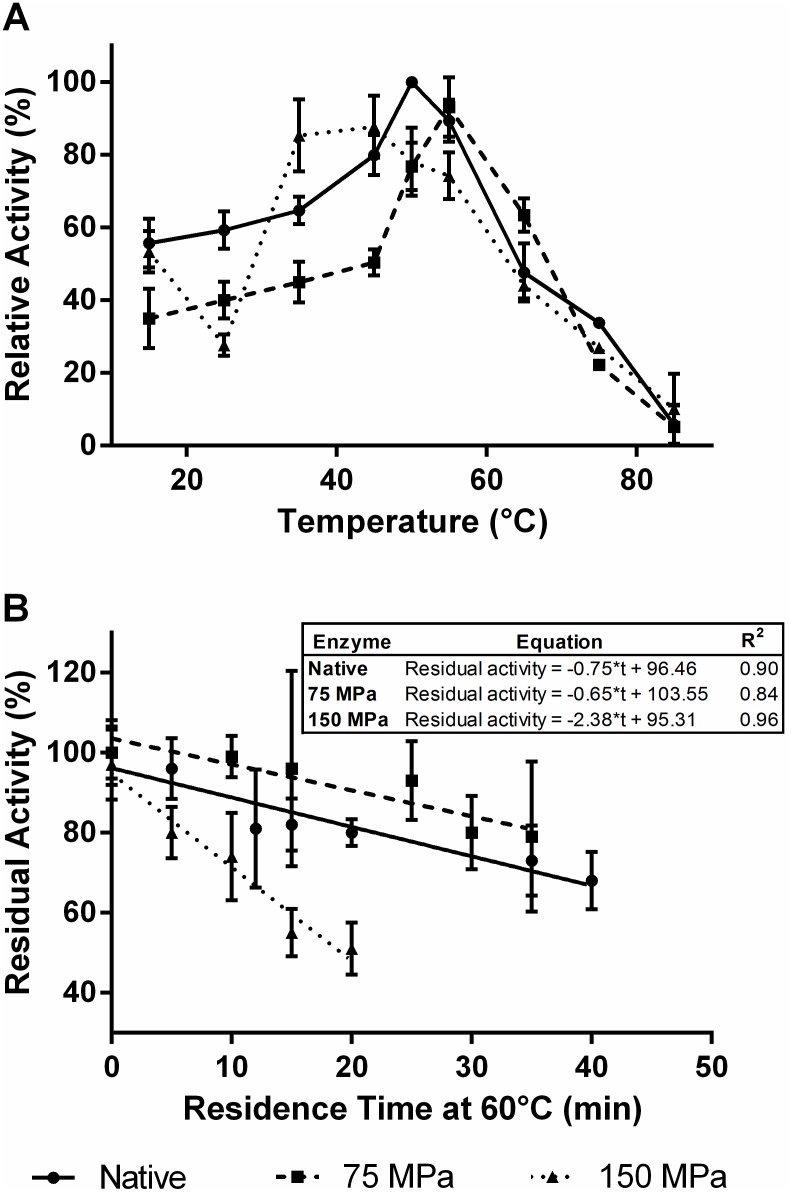
Effect of HPH treatment on the activity of GO at temperatures between 15 and 85°C (A) and in enzyme thermal resistance at 60°C (B).

Figure 2A illustrates the activity of the native and HPH enzymes measured at temperatures between 15 and 85°C. The data showed that after HPH treatment the temperature-activity profile of the enzyme changed in a HPH-dependent manner.

The native enzyme showed maximum activity at 50°C and it remained stable at lower temperatures with minimum activity of about 60% at 15°C. A typical enzyme deactivation was observed after the maximum point, reaching a minimum activity of 5% at 85°C.

The sample submitted to high pressure homogenization at 75 MPa showed less activity than the native enzyme at temperatures up to 50°C, and the maximum activity shifted to 55°C. However, the samples processed at 150 MPa showed an improvement in activity at lower temperatures, and the optimum activity shifted to 45°C. At temperatures above 55°C the enzyme activity showed similar behaviors for both the native and homogenized enzymes.

Although some differences were observed, in general the activity profiles of the homogenized and native enzymes were similar, indicating that the homogenization process was unable to roughly modify the activity of GO at different temperatures under the tested conditions. In addition, the only increment in GO activity was observed for the enzyme processed at 150 MPa and activity measured between 15 and 45°C. These results are different from those obtained for commercial GO, which showed a significant improvement in activity at 75°C after HPH [19]. It suggests that post-translational modifications in GO, such as glycosylation, produced under different environmental conditions are possibly related to distinct functional and stability behavior after HPH treatment.

### GO stability and kinetic parameters

The thermal stability of the enzyme was evaluated at 60°C for 40 minutes (Figure 2B). The results indicated that maintenance of the enzyme at 60°C caused a gradual and linear reduction in its relative activity. Figure 2B shows the linear models fitted to these data and the R^2^ values. According to these models, curves with similar slopes were obtained for the native and homogenized enzymes (75 MPa), indicating that 75 MPa was not sufficient to change the thermal stability of the enzyme. However, after HPH treatment at 150 MPa the slope of the curve increased around threefold, highlighting the fact that homogenization at this pressure considerably reduced the thermal resistance of GO. Other authors observed an increase in the thermal stability of trypsin after HPH at 80 MPa [24], demonstrating that different enzymes are differently affected by the HPH process.

**Figure 3:**
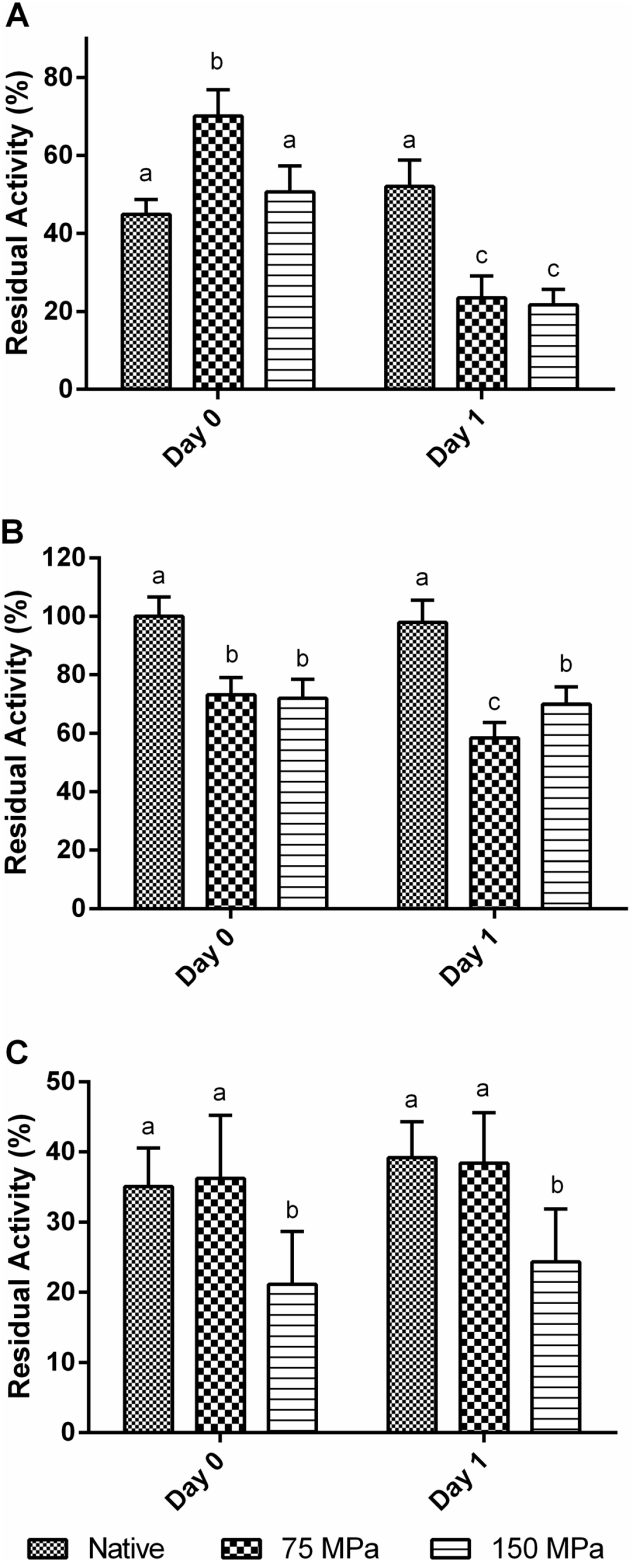
Activity of the native and HPH-treated enzymes determined (pH 5.0) at 15 (A), 50 (B) and 75°C (C) immediately after HPH treatment and after one day of refrigerated storage at 8°C.

The enzyme stability in aqueous solution at pH 5.0 was evaluated and the results are shown in Figure 3A (activity at 15°C), Figure 3B (activity at 50°C) and Figure 3C (activity at 75°C). The native enzyme showed no significant changes in the activity after one day at 8°C. For the homogenized enzymes, a reduction in activity was only observed at 15°C, and it remained the same activity level at 50 and 75°C. These results indicate that this enzyme is stable in solution and that HPH treatment did not change its stability. Previous results highlighted HPH as a process capable of improving the stability of a commercial GO, increasing the activity of the homogenized enzyme by between 100 and 400% [19]. Although those results showed an increase in enzyme stability, the enzymes were from different microbial sources and showed distinct primary sequences, which reflects in diverse thermal stability.

**Figure 4:**
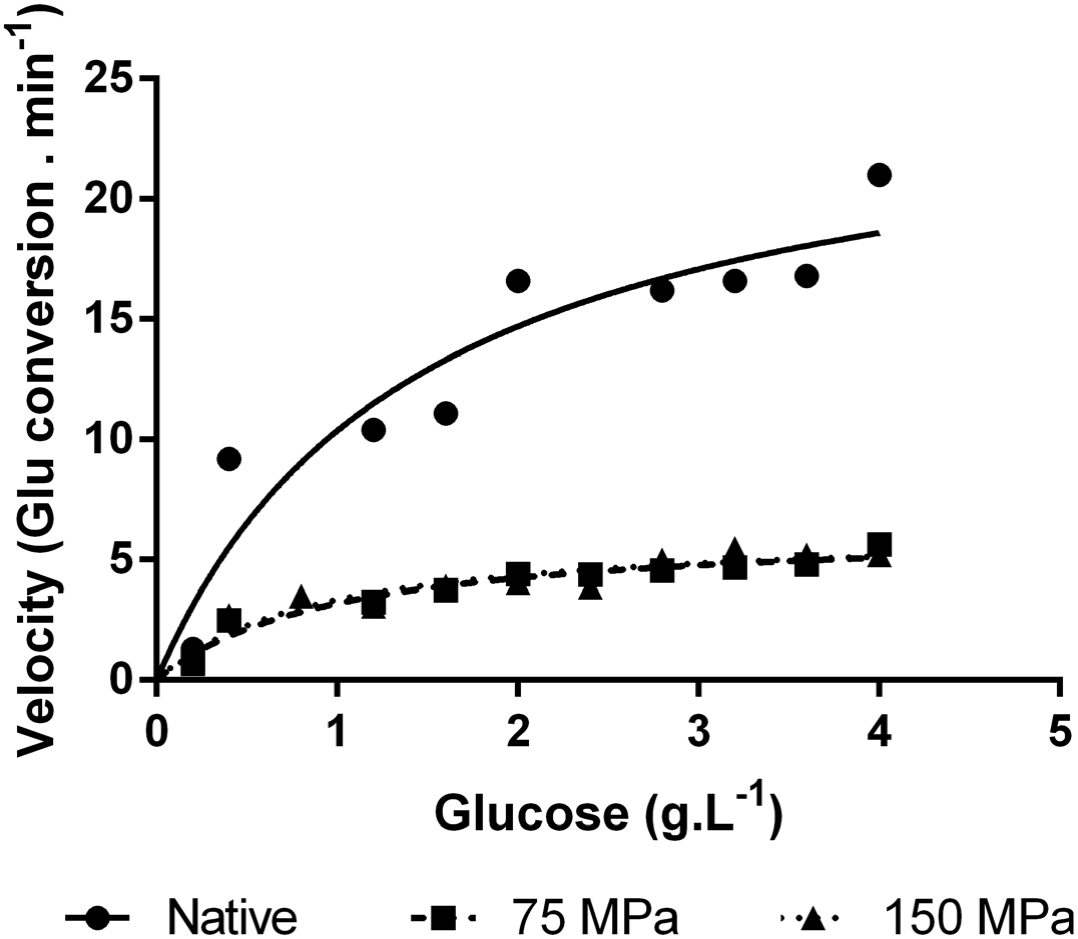
The effect of glucose concentration on the GO reaction velocity.

Figure 4 shows the curves obtained for the velocity of glucose conversion by the native and homogenized enzymes, measured using different substrate concentrations. Table 1 presents the kinetic parameters (V_max_, K_m_, k_cat_ and k_cat_/K_m_) obtained by fitting the data (Fig. 4) to the Michaelis-Menten model. The results showed that after GO HPH processing, the estimated K_m_-value was reduced by 30 and 40% in the samples homogenized at 75 and 150 MPa, respectively; and V_max_ was reduced fourfold for both homogenized enzymes when compared to the native enzyme. Thus homogenization produced an undesirable response in the velocity of GO at different substrate concentrations, suggesting that the homogenized enzyme became saturated at lower concentrations of substrate, as indicated by the K_m_ values. The analysis of the ratio between the parameters k_cat_ (turnover) and K_m_ (k_cat_/K_m_) suggested there was a reduction of about 60% in the catalytic efficiency of GO, with no difference between the different HPH treatments. This may be related to changes in the global conformation of the enzyme, which may alter the catalytic interface and consequently the recognition and binding of the substrate as noted by the inferior kinetic parameters.

**Table 1:** Kinetic parameters determined by the Michaelis- Menten model.

Kinetic parameter	Enzyme		
	Native	75 MPa	150 MPa
**V_max_ (µg/min)**	25.24	6.33	6.25
**K_m_ (g/L)**	1.44	0.98	0.87
**k_cat_ (s^−1^)**	0.02103	0.00528	0.00521
**k_cat_/K_m_**	0.01461	0.00538	0.00599
**R^2^**	0.88	0.94	0.88

The present findings suggest that the positive changes in the enzyme after HPH processing were mainly related to the increase in GO activity at neutral pH values. This improvement could be of interest when the GO is applied with the intention of reducing oxidation in products with neutral pH (milk, watermelon and melon juices) or to reduce the glucose content in liquid egg, aiming to prevent the undesirable browning caused by Maillard reaction [1], [4].

### Conformational changes associated to HPH treatment

**Figure 5:**
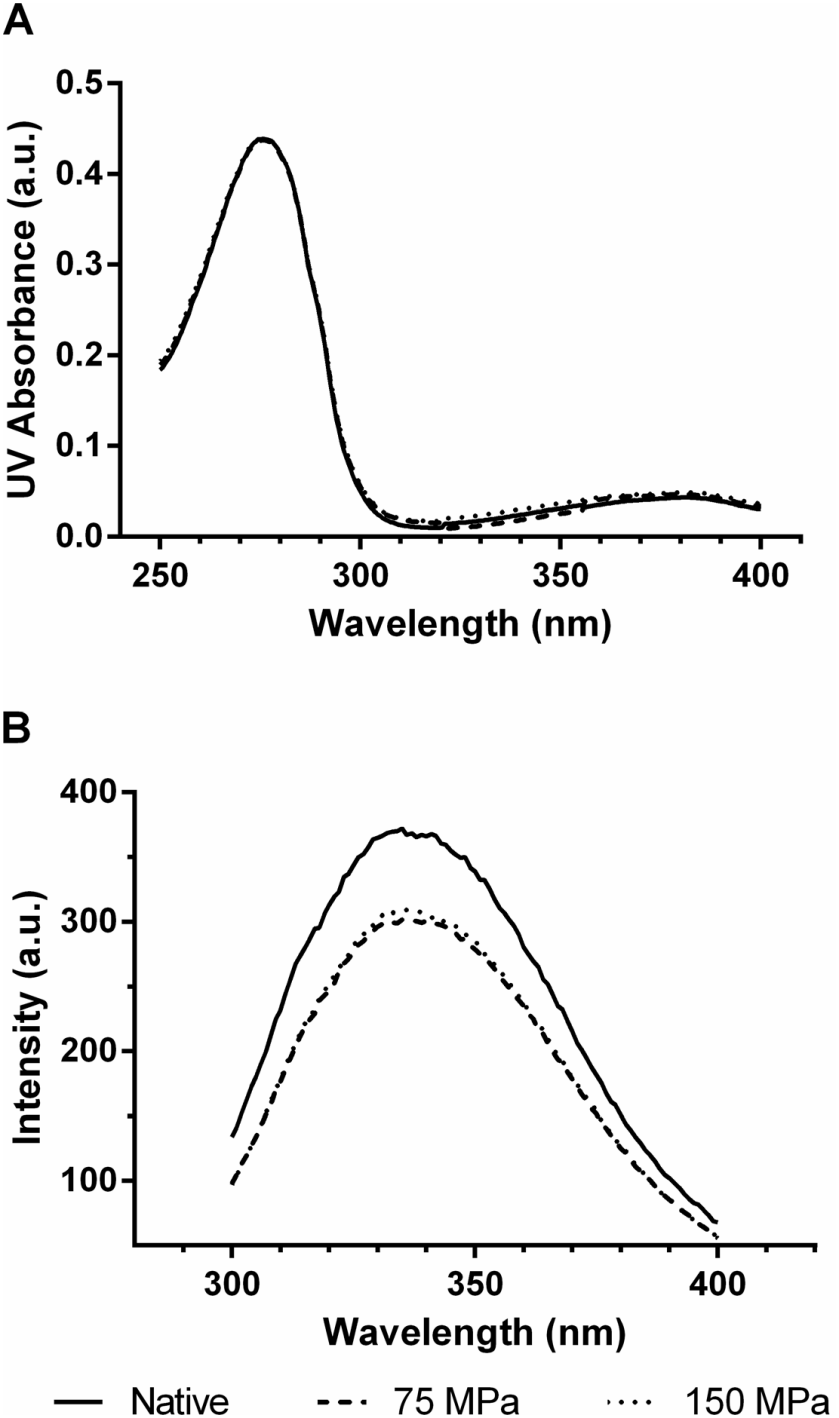
Effects of HPH treatment on GO conformation: UV absorption spectra (A) and Fluorescence emission spectra (B).

Spectroscopic experiments were carried out to evaluate the nature of HPH-induced modifications in GO structure. Figure 5A shows the UV absorption spectra of the native and homogenized GO samples. The UV absorption spectra were similar for the three samples, with a peak of absorbance at 275 nm. Typically, the disruption of secondary and tertiary structures of proteins is noticeable by shifts of the UV absorption maxima to shorter wavelengths and slight decreases in intensity [29], however by this method we were not able to detect the conformational changes induced by HPH treatment, suggesting minor or local modifications in the molecular structure.

Based on that, we analyzed the fluorescence emission maximum around 333 nm that is quite sensitive to local environment of tryptophane residues along with minor contribution from tyrosine. Indeed, Fig. 5B shows that the fluorescence emission spectrum of the native enzyme was distinct from that of the HPH-treated enzymes, highlighting conformational changes in GO upon HPH treatment.

**Figure 6:**
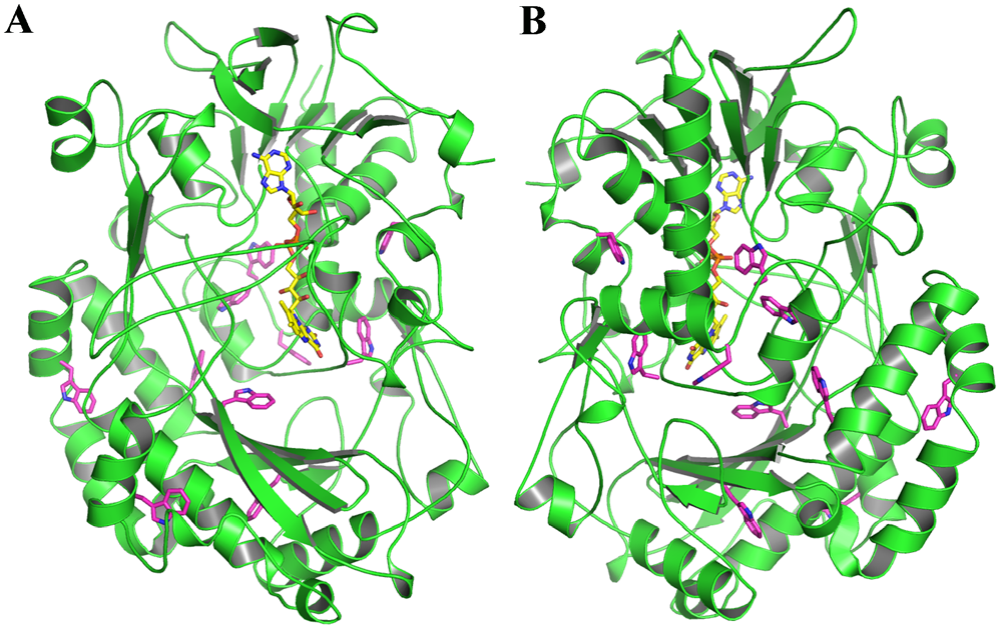
Schematic representation of the GO structure from *A. niger* (PDBID: 1CF3, Hecht et al., 1993) in two orientations (180°) indicating the positions of the tryptophan residues (carbon atoms in magenta). The FAD molecule is depicted in yellow.

HPH treatment reduced the fluorescence intensity by 19% at 335 nm and a red shift of the emission maximum from 335 to 336 nm was observed. Both events might be attributed to the exposure of the aromatic residues especially tryptophan to the solvent, which has a quenching effect [30]. Structural analysis of the GO structure from *Aspergillus niger* (PDBID: 1CF3, [31]) revealed several tryptophan residues located near to the surface, which could be easily exposed to the solvent by slight perturbations in the tertiary structure (Fig. 6A and 6B) supporting our spectroscopic results. The same effect on fluorescence emission was observed for other HPH-treated enzymes [16], [24], evidencing that HPH treatment effectively induces conformational changes in proteins.

**Figure 7:**
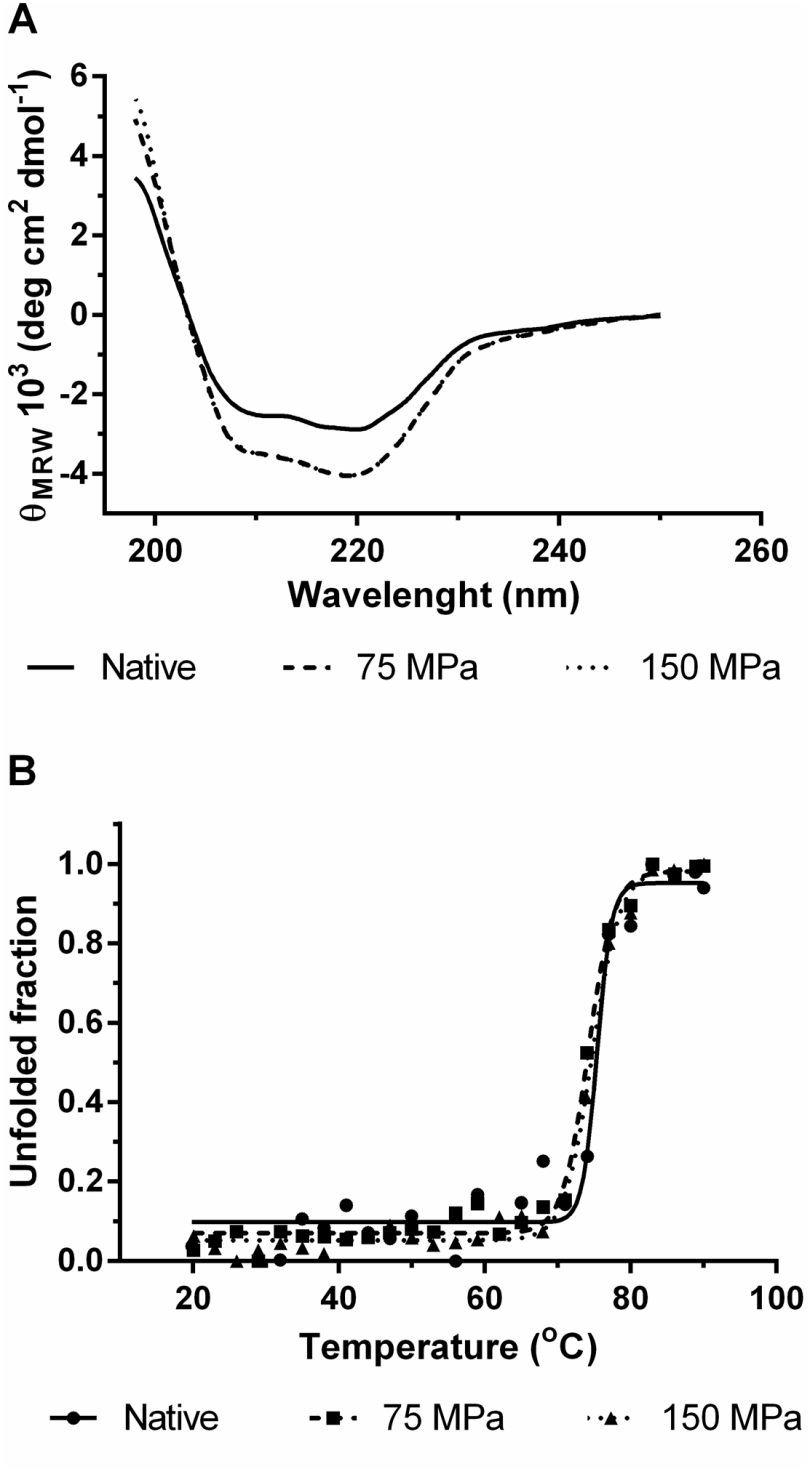
Circular dichroism analysis of GO secondary structure against HPH treatment: Far-UV CD spectra (A) and unfolding curves of GO (B).

We also investigated the effects of HPH treatment on secondary structure by monitoring circular dichroism spectrum in the far-UV range. As shown in Fig. 7A, both native and processed samples showed a characteristic CD profile of proteins with predominance of helical elements having two negative minima near to 208 and 220 nm. It is agreement with crystallographic structure in which 34% of the amino-acid residues (29 helices; 201 residues) are forming helices. In addition, CD analysis indicated that the protein underwent changes in its secondary structure by HPH treatment. An isodichroic point near to 203 nm along with the gain of the CD signal at 108 and 222 nm suggests a two-state dichroic model for a random-coil to α-helix transition [32] between the native and HPH-treated samples. It is likely associated with HPH-induced conformational changes as also demonstrated by fluorescence spectroscopy and since protein concentrations were precisely measured according to UV absorption analyses at 280 nm. Some authors have also reported that the HPH treatment was able to change the secondary structure, depending on the protein or enzyme [16], [24], [33].

We also determined the melting temperature of the native enzyme and processed at 75 and 150 MPa by monitoring the CD signal changes at 220 nm (Fig. 7B). These results show that although conformational changes did occurred in GO after HPH treatment, these structural rearrangements did not reflect on the thermal stability as assessed by loss of secondary structural elements. This observation is in agreement with the CD profiles of native and HPH-treated samples taking into consideration that these profiles did not indicate the disruption of secondary structure upon HPH treatment.

Collectively, these results establish that the HPH process promoted conformational changes in GO, and that these changes were nearly the same for samples treated at 75 and 150 MPa. In addition, it is likely that these structural rearrangements is associated with the distinct enzymatic behavior of GO that somehow affects the substrate recognition and consequently catalysis.

## Conclusions

The high pressure homogenization process promoted changes in the glucose oxidase activity, stability, kinetic parameters and structure. The treatment improved the activity of the enzyme at non-optimum pH values (4.5 and 6.5) or at low temperatures (15–45°C) after enzyme treatment at 150 MPa. For the majority of the tested conditions, the HPH-treated glucose oxidase presented similar responses, independent of the pressure used in homogenization. In addition, the biophysical characterization showed no differences between the HPH samples homogenized at 75 and 150 MPa, indicating that the structural rearrangements induced at 75 MPa led to a stable and new conformation of the molecule. Taking together these findings, we conclude that HPH treatment at 75 and 150 MPa changed the three-dimensional conformation of GO, affecting directly on the functional characteristics of the enzyme.
